# A split-mouth clinico-radiographic comparative study for evaluation of crestal bone and peri-implant soft tissues in immediately loaded implants with and without platelet-rich plasma bioactivation

**DOI:** 10.15171/joddd.2019.018

**Published:** 2019-08-14

**Authors:** Vande Aaditee Vishnu, Pronob Kumar Sanyal, Shivsagar Tewary, Kumar Nilesh, Roy Malvika Suresh Prasad, Karuna Pawashe

**Affiliations:** ^1^Department of Prosthodontics and Crown & Bridge, School of Dental Sciences, KIMSDU, Karad, India; ^2^Department of Oral and Maxillofacial Surgery, School of Dental Sciences, KIMSDU, Karad, India

**Keywords:** Immediate loading, one-piece implant, overdenture, platelet-rich plasma

## Abstract

***Background.*** This study evaluated the viability of platelet-rich plasma for enhancement of osseous and associated soft tissue healing around single-piece implants, subjected to immediate loading and to compare it with a control site not treated with PRP.

***Methods.*** Twenty completely edentulous patients were selected and 2 one-piece implants with O-ball head were placed for mandibular overdenture. The implant on the right side was treated with PRP whereas the left side implant served as a control. All the cases were immediately loaded and marginal bone loss, probing depth, percussion, implant mobility and peri-implantitis were assessed and compared at 3, 6, 9 and 12 months.

***Results.*** Overall analysis of the results showed less marginal bone loss, probing depth, percussion, implant mobility and periimplantitis around implants treated with PRP; however, the results were insignificant statistically.

***Conclusion.*** PRP can be used as a viable treatment adjunct in immediately loaded one-piece implants.

## Introduction

Branemark pioneered osseointegration in 1977.^1^ According to the earlier concepts of Branemark, a minimum healing period of at least 3–4 months was advocated, without loading in order to achieve osseointegration of dental implants.^2^ With time the understanding of physiology of bone healing at the implant‒tissue interface has changed. Various discoveries in implant therapy have paved the way for challenging the old preset protocols, replacing them with new ones. Developments in implant systems, designs, surfaces and more recently the use of bioactive platelet-derived growth factors have made it possible to shorten the healing time and improve implant success rate.

The advantages of implant-supported overdentures include ease to carry out oral hygiene procedures, control of denture movement and enhancing function and phonetics.^3^ Based on the 2002 MacGill Consensus Statement on overdentures, two-implant overdenture is considered the standard of care for edentulous patients.^4^ The standard two-piece implants cannot be used for severely resorbed cases. In such situations mini-implants can be used. One-piece dental implants have many benefits such as expanding the bone as they are placed, minimal osteotomy size required, immediate stabilization and loading on the day of placement and fewer treatment visits. Moreover, flapless placement leads to minimal surgical trauma, and easier removal and healing in case of failure. Their cost is also significantly less than the conventional implants.

Platelet-rich plasma (PRP) is an autologous source of growth factor which is obtained from freshly drawn venous blood. PRP has the ability to enhance and accelerate soft tissue repair and bone regeneration. The PRP applied to an implant surface adheres to metal and creates a new dynamic surface that potentially shows biologic activity. However, currently, there are very few human studies in the literature evaluating the clinical use of PRP in the bio-activation of one-piece implant surfaces for enhancement of peri-implant bone and soft tissue healing of immediately loaded implants. The aim of this study was to evaluate the viability of platelet-rich plasma for enhancement of osseous and associated soft tissue healing around single-piece implants, subjected to immediate loading and to compare it with a control site not treated with PRP.

## Methods

Twenty completely edentulous patients were selected for the study. A split-mouth study was designed. Two implants were placed in the mandibular arch for each patient for overdenture. The implant placed in the right quadrant was bioactivated with PRP (study site), whereas the left side served as a control site. One-piece 2.2×11.5-mm mini-implant with O-ball head (self-tapping, threaded root-form titanium-coated mini-implant, Genesis Implant, India) with O-ring housing (Metal housing with clear O-ring, Genesis Implant, India) were used for this study. Before implant placement, 5 mL of blood was drawn intravenously from the antecubital region of patients using flashback blood collection needle and BD vacutainer containing C.P.D.A. The vacutainer containing blood was centrifuged at 2400 rpm for 10 minutes. The result was separation of the whole blood into a lower red blood cell (RBC) region and upper straw-colored plasma containing platelet-poor plasma (PPP). PPP, buffy coat and upper 1-mm RBC layer were collected in a 12-mL borosilicate glass tube, counter-balanced and centrifuged at 3600 rpm for 10 minutes. The upper half of the supernatant was discarded and the lower half was mixed to yield PRP. PRP was transferred into a sterile stainless-steel bowl and 0.5 to 1 mL of 10% calcium chloride was added to PRP, leading to the formation of PRP gel.

All the aseptic precautions were taken. Once adequate anesthesia was achieved, a lancet drill was used for orientation of the osteotomy site through the patient’s mandibular denture placed in the mouth. The implant osteotomy site was prepared by a flapless technique on both sides in the mandible. On the left side, the implants were placed at the site without any PRP treatment. The implant to be placed in the right quadrant was dipped carefully in the PRP gel and immediately placed in the prepared site. An insertion torque value of 35 N.cm was achieved while placing implants on both sides.

Following the immediate loading protocol, the patient’s denture was modified to incorporate the metal housings with O-ring. Denture base resin from the intaglio surface of the denture was removed and the denture was allowed to passively fit against the tissue. A round bur was used to vent the pick up space toward the surface of the denture. A hole was punched in the center of two cut pieces of sterile glove, measuring approximately 2×2 cm. The glove pieces were pushed through the O-ball head of implants and placed over the ridge to avoid any thermal trauma to the mucosa while using auto-polymerizing resin in the patient’s mouth. The metal housings were fitted over the O-ball head of the implants on both the sides. The denture was thoroughly dried and petroleum jelly was applied on the portion of denture apart from the vent area. Auto-polymerizing acrylic resin powder and liquid were mixed according to manufacturer’s recommended ratio and filled into the vent created in the denture. The denture was placed intra-orally in position and the patient was asked to bite in the centric occlusion. The auto-polymerizing acrylic resin was allowed to set completely. Once the setting was complete, the denture was removed from the patient’s mouth and washed thoroughly. The excess acrylic material was trimmed and adaptation of the denture and patient comfort were assessed intra-orally. Occlusion of the dentures was rechecked in the centric relation position. Final finishing and polishing of the denture were carried out. Post-insertion instructions were given to the patient and insertion and removal of the denture were taught to the patient. The patient was recalled after 3 months for measuring the MBL on the mesial and distal aspects, respectively. The patient was subjected to radiovisiography (RVG) (Kodak, Carestream 5100). Digital radiographs were achieved using the paralleling technique. This technique was selected for the study so as to eliminate errors due to foreshortening or elongation or any other radiographic errors because of bisecting angle technique. To this end, RINN XCP positioning device was attached to the tube head of the x-ray unit. Digital radiographs were shot for implants on both sides. RVG special software for linear measurement was used to check for the MBL on the mesial and distal aspects of the implant and the readings were recorded.

Percussion test was used to assess osseointegration of the implants placed. The O-ball heads of the implants were subjected to vertical percussion with the metal handle of a mouth mirror. The following scores were characterized:

**Score 0:** crystal sound

**Score 1:** dull sound

Clinical assessment of the mobility of the implants was carried out by subjecting the O-ball heads of the implants to alternate pressure in a buccolingual and mesiodistal direction, using the handles of two mirrors. Any mobility was considered as failure of implant. Williams graduated probe was used to assess the probing depth around the implants. The probe is graduated at every 1 mm with markings missing at 4- and 6-mm measurements. The probing depth was assessed on the buccal, lingual, mesial and distal sides and the readings were recorded. A mean of all the 4 readings was calculated and this value was considered as the mean probing depth for that implant. All the implants were assessed for pain, inflammation, suppuration and bleeding around them. The implants which gave positive results for any of the above findings were considered failed. All these parameters were assessed at 3-, 6-, 9- and 12-month intervals and the readings were recorded for the implants treated with and without PRP in all the patients.

Data were recorded in Microsoft Excel 2010. The software used for statistical analysis was SPSS 17.

## Results

**Figure 1:**
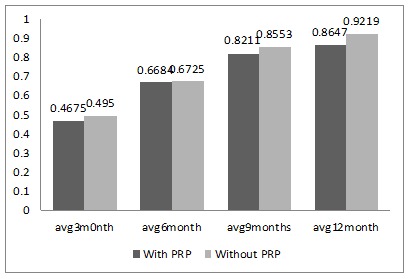
Mean overall marginal bone loss in mm at 3-month, 6-month, 9-month and 12-month time interval with and without PRP.
**Score 0:** crystal sound

The average overall marginal bone loss at 3-, 6-, 9- and 12-month intervals in groups with and without PRP are shown in Figure 1. The bone loss was greater in the group without PRP at all the time intervals. The mean marginal bone loss difference on the mesial side in the groups with and without PRP at 3-month interval was -0.03000, which was statistically insignificant (P=0.630). Similarly, at 6-, 9- and 12-month intervals these were also statistically insignificant (P=0.927, P=0.832 and P=0.160, respectively). For mean marginal bone loss difference on the distal side between the groups with and without PRP, similar statistically insignificant results were obtained (Table 1). When the mean probing depths, percussion results, mobility and peri-implantitis were assessed similar statistically insignificant results were found between the two groups.

**Score 1:** dull sound

**Table 1:** Comparison of mean marginal bone loss in mm at 3-, 6-, 9- and 12-month intervals on the mesial and distal aspects between the two groups (with PRP and without PRP) by using unpaired t-test

	**Mesial**			**Distal**		
	**Mean ± SD**	**Mean Difference**	**P-value**	**Mean ± SD**	**Mean Difference**	**P-value**
**With PRP (3 months)**	0.4400**±**0.20365	-0.03000	0.630	0.4950**±**0.15720	-.02500	0.615
**Without PRP (3 months)**	0.4700**±**0.18666			0.5200**±**0.15424		
**With PRP (6 months)**	0.6500 **±**0.30865	0.00789	0.927	0.6950**±**0.27429	0.00026	0.997
**Without PRP (6 months)**	0.6421**±**0.21684			0.6947**±**0.20405		
**With PRP (9 months)**	0.7947**±**0.23682	0.00789	0.832	0.8474**±** 0.25026	-0.05263	0.518
**Without PRP (9 months)**	0.8105**±**0.21831			0.9000**±**0.24721		
**With PRP (12 months)**	0.8471**±**0.09432	-0.05919	0.160	0.8824**±**0.07276	-0.05515	0.174
**Without PRP (12 months)**	0.9063±0.13889			0.9375±0.14549		

## Discussion

The one-piece implant design has an abutment attached to the implant itself, which makes it a single unit. Thus, it eliminates the microgaps between the abutment and implant. With all these advances in modern dentistry, the focus has shifted to decreasing the duration of implant therapy. To this end, three approaches have been developed: immediate, early and delayed loading.^5^ Immediate loading of implants shortens the overall treatment time and allows the patient to start using the prosthesis immediately after implant placement.

Another important factor to be considered during one-piece implant placement is the type of flap selected during the surgical procedure. Traditionally, the access for placement of implants has been through a flap. However, there are studies reporting that flap reflection often results in bone resorption around the natural teeth and the same goes with implants.^6^ Recently flapless technique has gained popularity in implant placement due to several advantages like a decrease in the postoperative discomfort, minimal bleeding at surgical site and no need for suture placement and reduction in surgical and healing time.^7^ Taking the above aspects into consideration, the present study was designed using one-piece implant with immediate loading through a flapless surgical procedure.

PRP has been shown to enhance bone and soft tissue healing and is widely used in several medical and dental procedures. PRP releases various growth factors, including PDGF, TGF, VEGF, epithelial growth factor (EGF) and insulin-like growth factor (ILGF).^8^ It also contains components such as fibrin, fibronectin and vitronectin, which are cell adhesion molecule and help in cell migration required for wound epithelialization and osseointegration. The release of PDGF, TGF and IGF initiates bone formation. VEGF promotes endothelial growth proliferation. EGF accelerates the epithelialization process and reduces scar formation. It also stimulates angiogenesis and collagenase activity.^9^ Due to the above advantages, PRP has become a promising adjunct treatment modality in implant dentistry.

Several animal and human studies have been conducted to assess the effect of PRP in implantology. Many of these studies have reported beneficial effects of PRP on hard and soft tissue healing around two-piece implants.^10,11,12^ However, in contrast to its known advantages, there are studies in the literature that do not show any significant benefit of using PRP.^13,14^ To the best of our knowledge, evaluation of the potential benefit of PRP on one-piece implants in overdenture cases have not been focused on. This study utilized a split-mouth design to assess the hard and soft tissue changes around one-piece implants with O-ball head in edentulous patients.

The parameters evaluated included assessment of marginal bone loss (MBL), probing depth, percussion results, implant mobility and presence of peri-implantitis. The parameters were studied every 3 months for a period of 1 year. The average MBL around the implants without PRP bioactivation was 0.9219**±**0.12776 mm, and around the implants bioactivated with PRP was 0.8647**±**0.07238 mm after a year. Similar results were reported by Elsyad et al,^15^ who compared MBL in conventional and immediately loaded implant-supported ball-retained mandibular overdentures. MBL of 0.91 mm was seen at 1-year follow-up in immediately loaded implants. Another study published in 2011 on immediately loaded mini-implants reported an MBL of 0.7‒1.2 mm after a year.^16^ A study by Jofre et al^17^ in 2010 showed a mean MBL of 1.4 mm around mini dental implants, which was higher than that in the present study. The MBL values on the mesial and distal sides were compared between the PRP and non-PRP groups, which showed less bone loss in the PRP group; however, the difference was statistically insignificant. This showed that treating implants with PRP did not have a significant benefit. Similar results were published by Malik et al^14^ in 2012 on mini-implants, where PRP did not show any significant difference among the study groups after 9 weeks. When intra-group comparisons of the average marginal bone loss were carried out between various time intervals, it was found that in the non-PRP group there was statistically significant bone loss at all the time intervals (3 months, 6 months, 9 months and 12 months). However, when similar comparisons were made in the PRP-treated implants there were statistically significant bone loss only between the time intervals of 3 and 6, 3 and 9, and 3 and 12 months. Intra-group comparisons between all the other time intervals showed insignificant bone loss. This suggested that for the PRP-treated implants, after 6 months of implant placement the average MBL was lower compared to implants without PRP treatment.

The mean probing depth after 12 months of implant placement was 1.5**±**0.36515 mm on the non-PRP treated implants, while 1.3529±0.42444 mm on the PRP-treated side. Elsyad et al^16^ reported a mean probing depth of 1.5**±**0.58 mm around immediately loaded mini-implants in their study, which was similar to the findings of the present study.The mean difference of probing depth in the PRP and non-PRP groups at 3-, 6-, 9- and 12-month intervals was found statistically insignificant. When compared within the groups, there was no significant difference between the probing depth values from the 3rd to the 12th month of follow-up in both groups. A possible explanation of this can be the design of one-piece implants that eliminates the micro-gap between the implant and the abutment, thus avoiding the violation of the biologic width.

Other clinical parameters included in this study were assessment of clinical mobility and peri-implantitis. Seven cases showed mobility, out of which 5 also showed peri-implantitis. Four implants which failed belonged to the non-PRP group, while three failed in the PRP group. On statistical analysis, this difference between the two groups was insignificant. When peri-implantitis was assessed, it was found that a total of 3 cases suffered from peri-implantitis. Out of these 3 cases two implants failed in one case, while for the other two cases one implant from the PRP group and one from the non-PRP group showed features of peri-implantitis. Hence the results were statistically insignificant.

A simple method to assess osseointegration of implants is the percussion test. A well osseointegrated implant gives a clear ringing crystal sound on percussion. A dull sound on percussion indicates improper osseointegration. In the present study the percussion test results were recorded according to the scale described by Batenburg et al.^18^ Seven implants (3 implants in the PRP group and 4 in the non-PRP group) had a score of 1 on percussion during the follow-up period, indicating failure. Statistical analysis showed insignificant difference of failure between the two groups.

All the failures that occurred in this study were after 6 months of implant placement. The implants came out in toto without any fracture. The possible reason for failure of implants in this study could be poor oral hygiene maintenance by the rural population who were candidates of the study. Marx et al^19^ and Lekovic et al^20^ reported that PRP accelerated the rate and degree of bone formation and soft tissue healing. An overall analysis of the parameters assessed in this study showed that although the results were insignificant for the PRP- and non-PRP-treated implants, the PRP-treated implants showed less bone loss and failure than the non-PRP-treated implants. Thus, we can conclude that PRP can be used as a beneficial adjunct to immediately loaded mini-implants.

## Conclusion

Shortening the long healing period is important for functional, esthetic and social well-being of the patients.^21^ Immediate loading of implants in such patients provides immediate functional and esthetic rehabilitation. This study was undertaken to evaluate the effect of immediate loading on mini-implants in overdenture patients. With the background that PRP has favorable effects on peri-implant hard and soft tissue healing, this study incorporated use of PRP as an adjunct in the implant therapy.

Irrespective of the implants treated with PRP, the overall success rate of the study was 82.5%, suggesting that immediate loading of one-piece implants is a viable treatment option for overdenture cases. Although treating with PRP resulted in less bone loss and probing depth around the implants during the 1-year follow-up period, the effect did not show any significant difference. This suggested that although adding PRP was beneficial, the effect was statistically insignificant.

With emergence of transfusion medicine science, the use of PRP is on the increase. With promising beneficial factors like the reduced cost, ease of PRP application in the dental clinic, biocompatibility of the material, autologous nature and the ready acceptance by the body, it has become a promising adjunct to dental surgical procedures as it provides safe and natural healing.

## Authors’ Contributions

VAV: principle investigator and preparation of manuscript, Contribution- principle guide. ST: clinical guidance for **cases. KN: clinical guidance for cases and review of** manuscript. RMSP: assisting in manuscript drafting. KP: assisting in manuscript drafting.

## Acknowledgments

The authors of this manuscript would like to acknowledge all the patients who agreed to participate in the study and also the statistician for interpreting the results.

## Funding

Not applicable

## Competing Interests

The authors declare no competing interests with regards to the authorship and/or publication of this article.

## Ethics Approval

Prior permission to initiate this research was obtained from the Institutional Ethical Committee. (PROTOCOL NUMBER-2016-2017/092).

## References

[1] Branemark PI, Hansson BO, Adell R, Breine U, Lindstrom J, Hallen O (1977). Osseointegrated implants in the treatment of the edentulous jaw Experience from a 10-year period. Scand J Plast Reconstr Surg Suppl.

[2] Branemark PI, Adell R, Albrektsson T, Lekholm U, Lundkvist S, Rockler B (1983). Osseointegrated titanium fixtures in the treatment of edentulousness. Biomaterials.

[3] Zarb GA, Schmitt A (1996). The edentulous predicament II: The longitudinal effectiveness of implant-supported overdentures. J Am Dent Assoc.

[4] Thomason JM (2002). The McGill consensus statement on overdentures Mandibular 2-implant overdentures as first choice standard of care for edentulous patients. Eur J Prosthodont Restor Dent.

[5] Yoo RH, Chuang SK, Erakat MS, Weed M, Dodson TB (2006 Mar-Apr). Changes in crestal bone levels for immediately loaded implants. Int J Oral Maxillofac Implants.

[6] Ramfjord SF, Costich ER (1968). Healing after exposure of periosteum on the alveolar process. J Periodontol.

[7] Siepenkothen T (2007). Clinical performance and radiographic evaluation of a novel singlepiece implant in a private practice over a mean of seventeen months. J Prosthet Dent.

[8] Simonpieri A, Del Corso M, Vervelle A, Jimbo R, Inchingolo F, Sammartino G, Dohan Ehrenfest DM (2012 June). Current knowledge and perspectives for the use of platelet-rich plasma (PRP) and platelet-rich fibrin (PRF) in oral and maxillofacial surgery part 2: Bone graft, implant and reconstructive surgery. Curr Pharm Biotechnol.

[9] Marx R (2004). Platelet-rich plasma: evidence to support its use. Journal of Maxillofacial surgery.

[10] Nikolidakis D, Van den Dolder J, Wolke JG, Jansen JA (2008). The effect of platelet-rich plasma on the early bone formation around Ca-P coated and non-coated oral implants in cortical bone. Clin Oral Impl Res.

[11] Zechner W, Tangi S, Tepper G, Fürst G, Bernhart T, Haas R (2003). Influence of platelet-rich plasma on osseous healing of dental implants: A histologic and histomorphometric study in minipigs. Int J Oral Maxillofac Implants.

[12] Anand U, Mehta DS (2012 Jan). Evaluation of immediately loaded dental implants bioactivated with platelet rich plasma placed in the mandibular posterior region: A clinico-radiographic study. J Indian Soc Periodontol.

[13] Weibrich G, Hansen T, Kleis W, Buch R, Hitzler WE (2004 Apr). Effect of platelet concentration in platelet-rich plasma on peri-implant bone regeneration. Bone.

[14] Malik A, Shaari R, Rahman SA, Aljuboori MJ (2012 Dec). Influence of platelet-rich plasma on dental implants Osseointegration in well-controlled diabetic patients. Dent Implantol Update.

[15] Elsyad MA, Al-Mahdy YF, Fouad MM (2012). Marginal bone loss adjacent to conventional and immediate loaded two implants supporting a ball-retained mandibular overdenture: a 3-year randomized clinical trial. Clin Oral Implants Res.

[16] Elsyad MA, Gebreel AA, Fouad MM, Elshoukouki AH (2011 Nov). The clinical and radiographic outcome of immediately loaded mini implants supporting a mandibular overdenture A 3-year prospective study. J Oral Rehabil.

[17] Jofre J, Hamada T, Nishimura M, Klattenhoff C (2010). The effect of maximum bite force on marginal bone loss of mini-implants supporting a mandibular overdenture: a randomized controlled trial. Clin Oral Implants Res.

[18] Batenburg RH, Meijer HJ, Raghoebar GM (1998). Treatment concept for mandibular overdentures supported by endosseous implants: a literature review. Int J Oral Maxillofac Implants.

[19] Marx RE, Carlson ER, Eichstaedt RM, Schimmele SR, Strauss JE, Georgeff KR (1998). Platelet-rich plasma: Growth factor enhancement for bone grafts. Oral Surg Oral Med Oral Pathol Oral Radiol Endod.

[20] Lekovic V, Camargo PM, Weinlaender M, Vasilic N, Kenney EB (2002). Comparison of platelet-rich plasma, bovine porous bone mineral, and guided tissue regeneration versus platelet-rich plasma and bovine porous bone mineral in the treatment of intrabony defects: a re-entry study. J Periodontol.

[21] Saleem M, Saleem R, Meshack RA, Guru RC (2011). Prosthetic management of edentulous mandible using endosseous implants and overdentures. J Contemp Dent Pract.

